# Address before the Graduating Class, in the Ohio College of Dental Surgery, March 1, 1871

**Published:** 1871-06

**Authors:** Jas Taylor

**Affiliations:** President of Board of Trustees


### ADDRESS BEFORE THE GRADUATING CLASS, IN THE OHIO COLLEGE OF DENTAL SURGERY, March 1, 1871.

BY DR. JAS. TAYLOR, PRESIDENT OF BOARD OF TRUSTEES.

GENTLEMEN GRADUATES:

By examination of your diplomas you will find a very significant motto on the seal thereof. It is this: "Science Combats Disease." Disease is represented as an aggressive, insiduous foe of our race, ever at work to undermine our health and lay us prostrate at her feet. She comes to us hidden in the food we eat, in the water we drink, and the invisible air we breathe, often comes laden with her pestilential influence. Our recreations, our amusements and pleasures, all must be kept within proper bounds, or this enemy is upon us. Even too much sleep, the great restorer of exhausted nature, may open the door for this enemy to step in.

Science is represented as an ever watchful guardian—fortifying us at this and that point to resist attack. Showing when and how to avoid her approach, and when the enemy is upon us placing means and weapons in our hands by which to drive off disease. I take it, there is a continuous battle going on between medical science and disease, and although disease, from the nature of things, does ultimately triumph, yet the victory is often deferred and pain and suffering immensely mitigated. If there is therefore a truly noble pursuit, it is that which has for its object and aim the prolongation of life. It is noble because it teaches us what is man in all his mysterious organization, the most noble of God's creation. Science explains the laws and functions of life. Its study not only enlarges our views of man, but reveals much of that benificent wisdom which brought him into being.

We take simply dentition from its earliest developmental process unto its consummation, and what a study it affords! the laws which govern accretion; the elements necessary for perfect dental development; the power and office of the secreting vessels; the wonder that so hard a body should be elaborated from the circulating fluid. The wonderfully mysterious apparatus by which the deciduous organs are destroyed, and the wisdom of their destruction and replacement. The whole process, from germ life to perfection, should forever silence the skeptic's argument of *chance.*

In the prosecution of your studies we have no doubt you have been led to admire the laborious and persistent research which has so fully demonstrated these facts of our science, and as they are the foundation principles of dental physiology, we would urge upon you their careful consideration.

The first duty of the physician is to prevent disease. These laws of our economy point with unerring certainty to the only means of securing perfect development of our dental organs. An imperfect organization can be expected to resist but feebly the varied causes producing disease.

Help, then, gentlemen, to turn back our artificial mode of living to that natural, normal condition when decay of the teeth was as rare as it is now common. Without this change all our skill and care can only retard and partially preserve. It is a sad reflection that so many Americans need the services of the dentist, and we may add, it is a proud reflection that so much good is accomplished through their instrumentality. Yes, gentlemen, "Science does Combat Disease," and our profession is making a proud record for herself. We bid you welcome to her ranks, and as your opportunities have been good and you have shown commendable zeal in availing yourselves of them, we shall look to you with confidence that her march shall be onward; that future progress shall be in correspondence with the past, and your names be all enshrined in the hearts of the profession.

A few of us can look back professionally to the time when there was not a dental college in the land, and by this we mean that perhaps not in the world. The old world as well as the new had not as yet recognized dentistry as a science, even as an honorable art it was struggling for recognition. A few whose names will always stand as stars in the dental galaxy were silently but faithfully making their mark on this continent, but still a French name was almost only relied upon for a recommendation. This was then the diploma, and brought with it succedaneum and a train of French evils; but now what a change! Paris and many of the capitals of Europe look to America for dental skill, and your diplomas are a passport thoughout the civilized world. They will give you introduction to the medical fraternity wherever medical science is recognized as a liberal profession. They are evidence of an examination here, which courtesy affirms is sufficient even where the most strict laws are passed to guard every specialty of medical science. They are worthy, then, of your honor. Strive to make those of your Alma Mater the par excellence of all the honored.

But, gentlemen, the seal on your diplomas reveals something more than the motto to which we have referred. There is the sun shedding its refulgent light on an open book, symbolic, if you please, of the immense store of knowledge which science offers to the student. The book is open and God's pure light shines upon it, that all may read and understand. The book is not a shut and sealed one, hid away in the dark. It is a book of "knowledge of good." Yes, it is a good book, and every fact or principle of science it contains reveals more and more the infinite wisdom of the Author of all true science.

We do not wish to be understood as meaning that all books contain nothing but what is good, for such is not the case. Yet all the facts of science will be found so far as yet discovered in books, and to them you must go for knowledge. We must learn to sift the good from the bad; to take facts as demonstrated, and not theories for our guide.

Books should become now more than ever your constant companions. You select the best authors, read them with care, and you will soon find that your preparatory studies have but prepared you for the full force and proper appreciation of what you read. This is really your commencement, but commencement of what? It is the commencement of your professional career, and we had almost said the commencement of your studies, and why should it not be? If you live for years to come, it should be but the commencement of your knowledge. We are glad to learn that you have made laudable proficiency; a solid foundation for future attainment and usefulness. Continued study will alone secure this.

We would not have you confine your reading to that which relates directly to the profession. You should take in all science, morals and philosophy, and let me here say that the best of all books on morals is the Bible, which this institution has always recognized as the book of books, and all our graduates have been presented with a copy. Keep yourselves thoroughly posted in your profession. But you will find that to diversify your reading your mind is relieved, enlarged, expanded, so that your regular studies will be attended with more zest. Recollect that our periodical literature is always rather in advance of our standard works. New dental books are and must necessarily be very much made up from selections and compilations from our periodicals. They appear to be only necessary, as new and valuable information is thus accumulated, requiring the gathering up of this rich material and condensing and systematizing it for convenient use.

No dental or medical office can afford to dispense with the new living thought and experience which comes fresh and sparkling every month or quarter from the press.

These are the channels, gentlemen, through which we hope to see results of your experience and observations, not alone of books, but the working out of your own thoughts? Think for yourselves and make books and observation the fuel for your thoughts.

"Science Combats Disease." Read it not that ignorance combats disease; disease may be relieved ignorantly; accidents happen occasionally. Ignorance vaunts itself; it is puffed up; it is full of pride and high sounding words. If you doubt this read the advertising page of any daily paper. This is all not even false science; there is no science about it; it is empiricism. True science comes to the student, to the humble seeker after truth, as an analyzer of facts, an investigator of principles, a friend of every devotee of knowledge.

In conclusion, gentlemen, let me say that in some way or other she will always be found of those who seek her.

